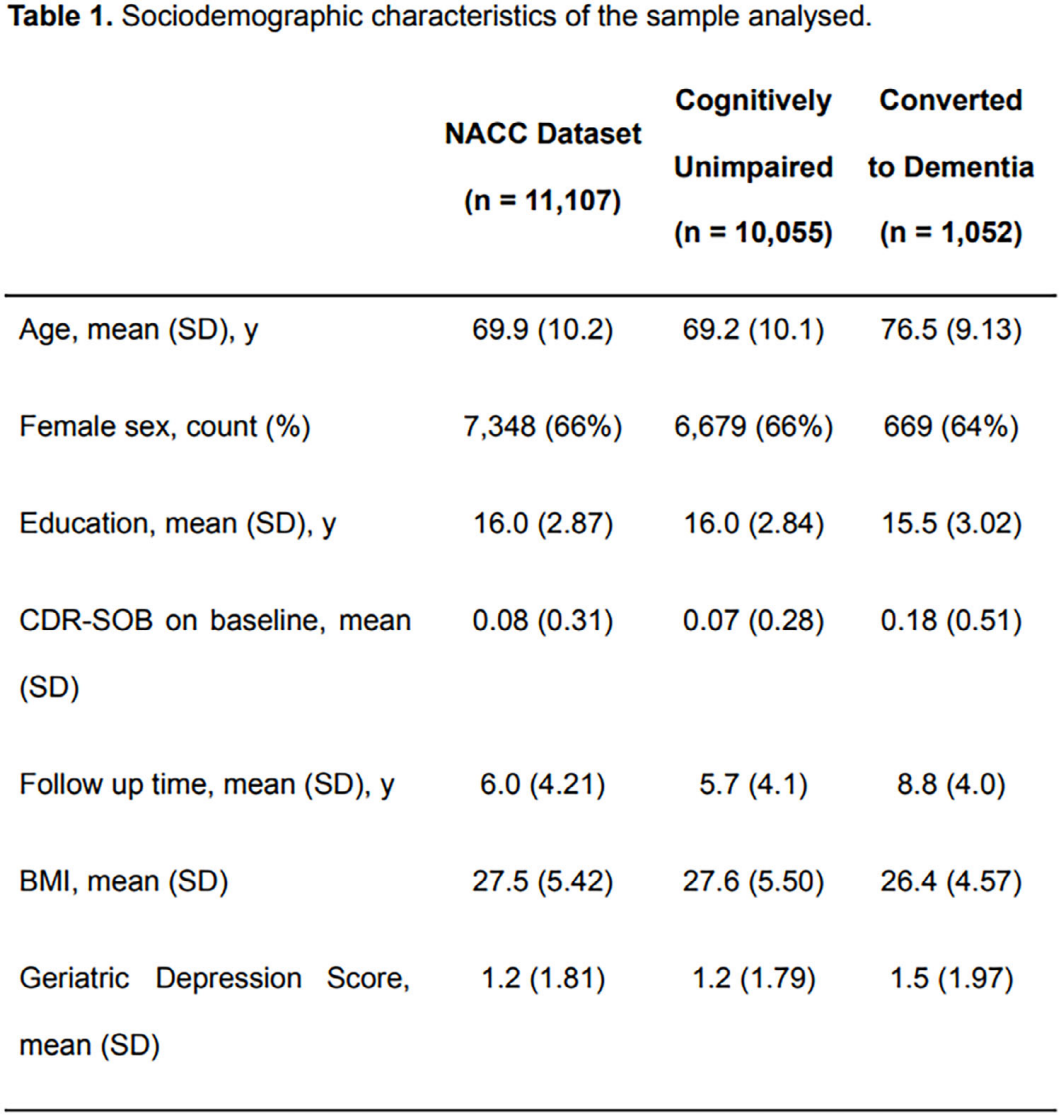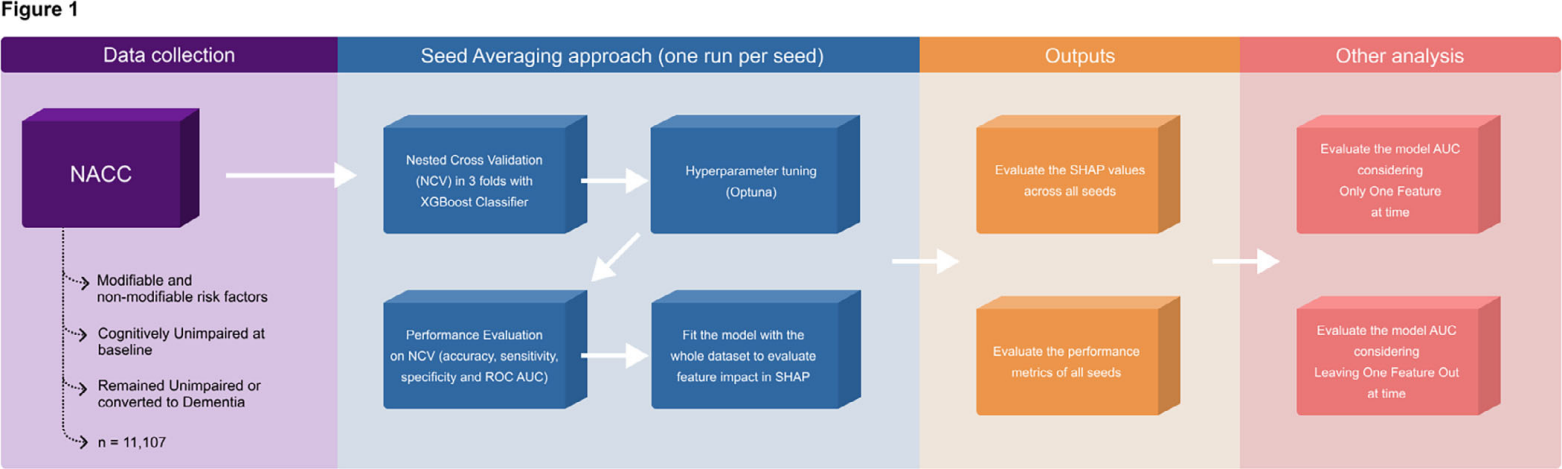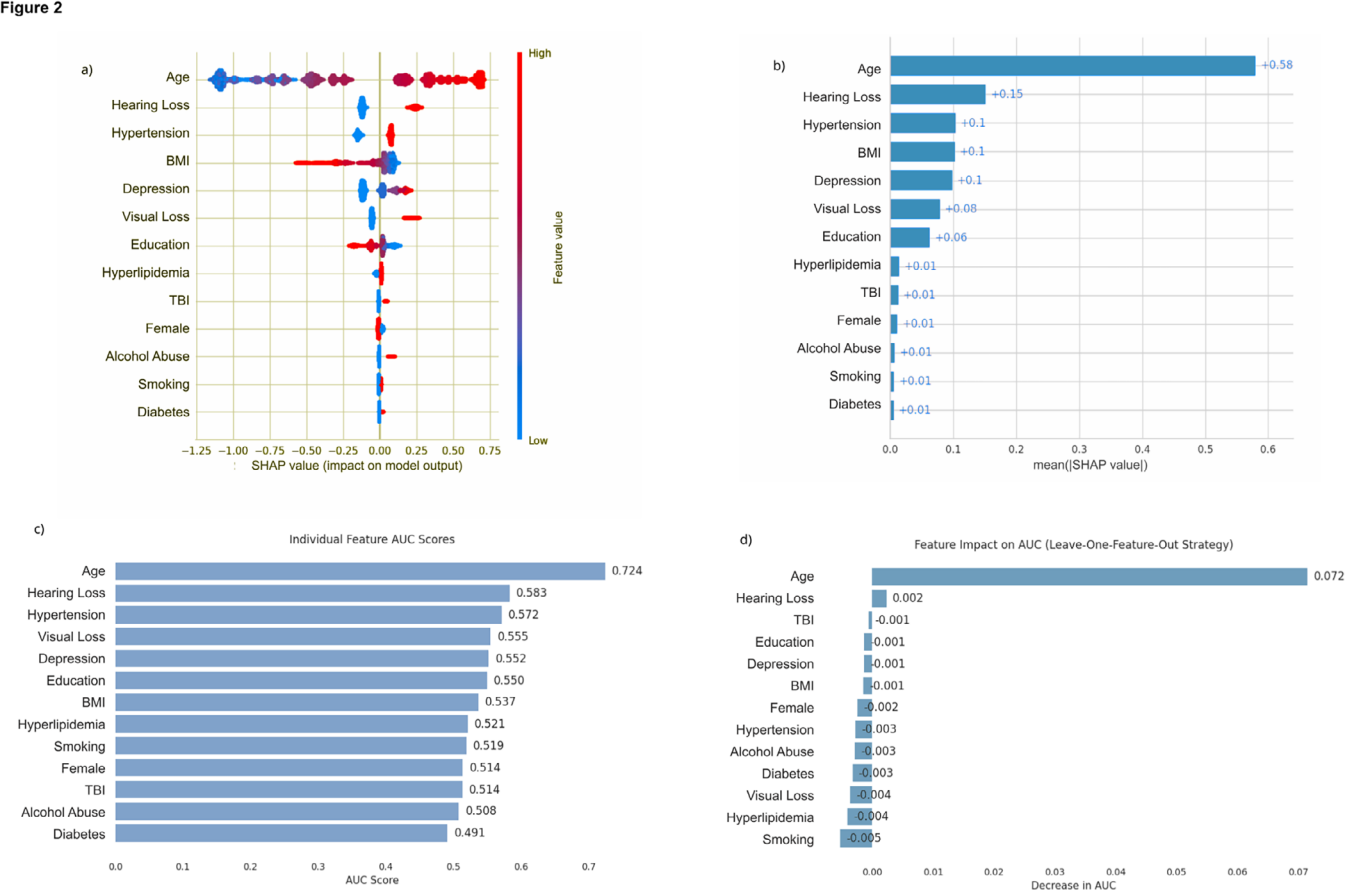# A Stratified Machine Learning Evaluation of Risk Factors of Dementia Conversion

**DOI:** 10.1002/alz70860_105523

**Published:** 2025-12-23

**Authors:** Daniel Arnold, João Pedro Ferrari‐Souza, Rodrigo C. Barros, Marco De Bastiani, Eduardo R. Zimmer, Wyllians Vendramini Borelli

**Affiliations:** ^1^ Universidade Federal do Rio Grande do Sul, Porto Alegre, Rio Grande do Sul, Brazil; ^2^ Universidade Federal do Rio Grande do Sul, Porto Alegre, RS, Brazil; ^3^ PUCRS, Porto Alegre, Rio Grande do Sul, Brazil; ^4^ McGill University, Montreal, QC, Canada; ^5^ Brain Institute of Rio Grande do Sul ‐ Pontifícia Universidade Católica do Rio Grande do Sul, Porto Alegre, Rio Grande do Sul, Brazil; ^6^ Centro de Memória, Hospital Moinhos de Vento, Porto Alegre, RS, Brazil; ^7^ Clinical Hospital of Porto Alegre, Porto Alegre, Rio Grande do Sul, Brazil; ^8^ Brain Institute of Rio Grande do Sul (InsCer), PUCRS, Porto Alegre, Rio Grande do Sul, Brazil

## Abstract

**Background:**

Understanding the complex interplay of risk factors for dementia is essential for developing effective prevention strategies. Older adults present a high frequency of multimorbidity, though risk factors of dementia are usually evaluated individually in this population. In this study, we aim to simultaneously identify modifiable and non‐modifiable risk factors in predicting dementia conversion.

**Method:**

Real‐world longitudinal data from the National Alzheimer's Coordinating Center (NACC), spanning 2005 to 2023 across 46 Alzheimer's Disease Research Centers (ADRCs) was analysed. Eleven modifiable risk factors were stratified: hearing loss, hypertension, body mass index (BMI), depression, visual loss, education, hyperlipidemia, traumatic brain injury (TBI), alcohol abuse, smoking, and diabetes. Age and gender were analyzed as non‐modifiable factors. A machine learning approach was employed for simultaneous evaluation of risk factors (Figure 1).

**Result:**

We included 11,107 cognitively unimpaired individuals at baseline, whose 1,052 converted to dementia (Tab. 1). SHAP (SHapley Additive exPlanations) value analysis assessed the impact of each factor, with Figure 2a displaying the distribution of impacts and Figure 2b illustrating their absolute contributions. The overall performance of the model included an average accuracy of predicting dementia conversion of 66.03% (95% CI: [65.82, 66.23]), sensitivity of 70.26% (95% CI: [70.03, 70.48]), specificity of 65.59% (95% CI: [65.34, 65.83]), and an ROC‐AUC of 0.745 (95% CI: [0.744, 0.745]). Age was the most impactful, with an individual AUC score of 0.724 (Figure 2c), and a strong influence on model performance (Figure 2d). Hearing loss, hypertension, BMI, depression, visual loss and education emerged as impactful modifiable risk‐factors in our model, albeit not as much as age.

**Conclusion:**

This study highlights the significance of a simultaneous evaluation of risk factors of dementia, considering multimorbidities in dementia prevention. While age is a primary predictor, our approach identifies critical points within modifiable factors like hearing loss and hypertension. The machine learning framework enhances predictive accuracy, offering comprehensive insights for prevention strategies. Future studies should focus on validating these findings across diverse populations and longitudinally.